# The prevalence and characteristics of suicidality in HIV/AIDS as seen in an African population in Entebbe district, Uganda

**DOI:** 10.1186/1471-244X-12-63

**Published:** 2012-07-12

**Authors:** Eugene Kinyanda, Susan Hoskins, Juliet Nakku, Saira Nawaz, Vikram Patel

**Affiliations:** 1 MRC/UVRI Uganda Research Unit on AIDS & Senior EDCTP Fellowship, P.O. Box 49, Entebbe, Uganda; 2 Medical Research Council, Clinical Trials Unit, London, UK; 3 Butabika National Psychiatric Referral Hospital, Kampala, Uganda; 4 Dartmouth Institute, Dartmouth College, Hanover, USA; 5 London School of Hygiene & Tropical Medicine, UK & Wellcome Trust Senior Research Fellow in Clinical Science, London, UK

**Keywords:** HIV/AIDS, Suicidality, African population, Attempted suicide, Prevalence

## Abstract

**Background:**

Suicidality in HIV/AIDS is not only a predictor of future attempted suicide and completed suicide, it is also associated with poor quality of life and poor adherence with antiretroviral therapy. This paper examines the prevalence and correlates of suicidality in HIV/AIDS in the African nation of Uganda.

**Methods:**

A cross-sectional study was undertaken among 618 respondents attending two HIV clinics in semi-urban Uganda. A structured questionnaire was used to collect data on demographic, social, psychological and clinical factors. Correlates of suicidality were assessed using mulitvariable logistic regression.

**Results:**

Prevalence of ‘moderate to high risk for suicidality’ (MHS) was 7.8 % and that of life-time attempted suicide was 3.9 %. Factors associated with MHS at univariate analysis were: female gender, food insecurity, increasing negative life events, high stress score, negative coping style, past psychiatric history, psychosocial impairment, diagnoses of post-traumatic stress disorder, generalised anxiety disorder and major depressive disorder. Factors independently associated with MHS in multivariate models were female gender, increasing negative life events, a previous psychiatric history, and major depressive disorder.

**Conclusions:**

These results are in agreement with the stress-vulnerability model where social and psychological stressors acting on an underlying diathesis (including previous and current psychiatric morbidities) leads to suicidality. These results identify potential targets to mitigate risk through treatment of psychiatric disorders and promoting greater adaptation to living with HIV/AIDS.

## Background

Suicidality, which for purposes of this study includes both significant suicidal ideation and attempted suicide, is one of the psychiatric problems associated with HIV/AIDS [1]. A consideration of suicidality in HIV/AIDS is important not only because it predicts future attempted suicide and completed suicide, it has also has been associated with poor quality of life, poor adherence with ART (antiretroviral therapy) and non-disclosure of HIV status to significant others [1,2]. The few African studies on suicidality in HIV/AIDS have reported the following prevalence rates: 12.4 % for suicidal ideation among patients attending a specialized HIV/AIDS clinic in pre-ART Uganda; 17.1 % for the 12 month prevalence of attempted suicide rate among HIV positive adolescent in pre-ART Uganda; 13 % for current suicidal ideation among patients attending a specialized HIV/AIDS clinic in post-ART Uganda; and 16.8 % for suicidality among HIV positive patients in South Africa [3-6]. All these studies reported on the prevalence of suicidality as a secondary finding, with none reporting on the correlates of suicidality.

Studies in the west have reported the following risk factors for suicidality in HIV/AIDS: socio-demographic factors (female gender, younger age, black ethnicity); psychiatric problems (substance abuse, major depressive disorder, antisocial personality disorder, previous attempted suicide, family history of a psychiatric disorder, family history of attempted suicide); psychosocial factors (heterosexual orientation, multiple HIV related loses, lack of social support, loss of employment or insurance cover, exhaustion of financial resources, physical and sexual abuse); and clinical factors (painful and disfiguring physical deterioration, suffering from lipodystrophy-related symptoms, physical and psychological symptoms, AIDS diagnosis) [1,7-13]. In the only published African study to date that has looked at risk factors for suicidality in HIV/AIDS, Schlebusch and Vawda (2010) in South Africa reported the following risk factors: female gender, psychiatric disorders (major depressive disorder and substance abuse), partner relational problems, poor social support, fear of disclosure/stigmatization, socio-economic pressures, cognitive deficits (problems with cognitive flexibility, concentration and memory).

This paper examines the prevalence and correlates of suicidality in HIV/AIDS as seen in the African socio-cultural context of Uganda with a view to inform the development of mental health interventions for persons living with HIV/AIDS (PLWHA) on the African sub-continent.

## Methods

### Study design

This was cross-sectional study undertaken at two HIV clinics in the semi-urban district of Entebbe, Uganda. All consenting eligible HIV-infected patients attending the government health facilities of Entebbe District Hospital and Kigungu Health Centre III were continuously enrolled into this study. To be eligible for this study, the individual must have been registered with the study HIV clinics, 18 years or older, fluent in English or Luganda (the local language into which the study instruments had been translated) and not so physically and mentally sick as to be unable to complete the interview. Trained psychiatric nurses recruited from Uganda’s National Psychiatric Referral hospital at Butabika conducted structured interviews to determine the prevalence and correlates of psychiatric disorders among the respondents.

### Data collection tools

The data collection tool for the cross sectional study component consisted of various structured and standardized modules which were translated into Luganda (the local language spoken in the study area) and were administered by trained psychiatric nurses, these included:

1) Socio-demographic factors sex, age, marital status, highest educational attainment, religion, and employment status.

2) Social factors i) ‘ (assessed by the question, *‘in the last month, did you or your family have enough food?’*); ii) ; iii) ; iv) ‘ , constructed from items of the adverse life events module of the European Parasuicide Interview Schedule [14] that has previously been modified for the Ugandan situation by Kinyanda and colleagues (2005a) [15]. For this study, respondents were asked whether they had experienced each of these events in the last 6 months. Items were selected for inclusion in this study based on the relevance to the HIV social situation in Uganda. Items were included to reflect the key social relationships in an individual’s life, namely parent (5 items), sibling (6 items), spouse/lover (5 items), child(ren) (4 items) and the individual (7 items) with questionnaire items such as *‘did your father die?’* and *‘have you been very ill ?’.* A total score was generated to reflect the total number of life events reported, this scale had an α Cronbach of 0.82 in this study; v) ‘ , constructed by scoring each of the reported negative life events on a 3 point Likert scale where respondents were asked the question, *‘how stressful did you find the event?’* with possible responses being: 0 = (not stressing/minimal stressing), 1 = (moderately stressing), 2 = (severely stressing). A total score was generated where high scores reflected more stress; vi) ‘ , constructed from items of the social support module of the European Parasuicide Interview Schedule [14]. This scale has four sub-scales each with 4 items that assess the following social support dimensions: ‘need for social support’, ‘receive the required social support’, ‘felt needed for social support’ and ‘felt was providing the required social support’. Each of the items in these sub-scales is scored on a 3 point Likert scale where 1 = (not at all), 2 = (to some extent), and 3 = (yes, very much). Items of the sub-scale ‘need for social support’ were reverse scored so that higher scores on this indicated better social support just like the other three sub-scales. A total score was generated with higher scores reflecting better social support, the α Cronbach of this scale in this study was 0.84.

3) Psychological and clinical factors i) was assessed using the M.I.N.I. neuropsychiatric interview (MINI Plus) [16] which is a modular DSM IV based structured interview. The psychiatric disorders modules used in this study were suicidality, major depressive disorder (excluding the suicidality items), alcohol use disorders, generalised anxiety disorder and post-traumatic stress disorder; ii) ‘ was constructed from variables of the Mental Adjustment to Cancer Scale (MAC) [17] whose items had been adapted to the local HIV situation. Each of this scale’s 17 questionnaire items is scored on a 4-item Likert scale 1 = (definitely does not apply to me), 2 = (does not apply to me), 3 = (applies to me), 4 = (definitely applies to me). To score all the questionnaire items so that they are all in the same direction i.e. higher scores reflecting more negative coping style required that questionnaires items (1,4,5,10,11,12, 13,15,16) that are cast positively such as item 1: *‘I have been doing things that I believe will improve my health* e.g. *changed my diet.’* be reverse scored. A total score was generated so that higher scores reflected a more negative coping style, the α Cronbach of this scale in this study was 0.58; iii) ‘ was assessed by the question, *‘have you ever suffered from any nervous or psychiatric condition?*); iv) ‘ ; v) ‘ ’ assessed using the International HIV Dementia Scale [18]; vi) ‘ constructed from the three variables *‘in the last month, on how many days were your normal activities disrupted through illness?’* (with responses scored as follows: none = score 0, one and above = score 1); *‘how many times did you visit the health unit in the last month?’* (with responses scored as follows: none = score 0, one and above = score 1), *‘for how many days were you admitted to hospital in the last month?’* (with responses scored as follows: none = score 0, one and above = score 1). A total score was generated so that higher scores reflected more impairment, this scale had an α Cronbach of 0.54; vii) ; and viii) .

### Statistical analysis

**Figure 1:**
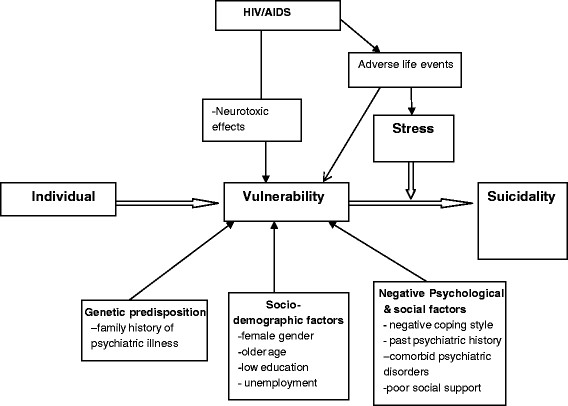
**Conceptual framework based on the Stress-Diathesis Model (Mann et al., 1999) **[19]

A conceptual framework (Figure 1) based on the stress-vulnerability model for suicide was specified a priori to guide the multivariate analyses and to avoid the problems of colinearity [19]. Statistical analysis was undertaken using both SPSS (reliability tests) and STATA. Logistic regression models were used to assess univariate associations between the dependent variable ‘moderate to high risk for suicidality’ (MHS) and independent variables, grouped in sets of demographic, social and psychological risk factors, with unadjusted and adjusted Odds ratio (adjusted for sex and age group, as a priori confounders) reported. The dependent variable in this paper is ‘moderate to high risk for suicidality’ (MHS) defined as a score of 9 and above on the B-items of the suicidality module of the M.I.N.I. neuropsychiatric interview (MINI Plus). The constituent items of the suicidality module of the M.I.N.I included items that assessed for previous attempted suicide, suicidal ideation and feelings of hopelessness. The rest of the investigated factors were considered as the independent variables.

The selection of the final model was done in stages, at each level of the framework. Variables whose age- and sex-adjusted (as a priori confounders) association with the outcome was significant at p < 0.10 were added to the model one by one, and those remaining associated at p < 0.10 were retained. Thus, social risk factors were considered one at a time, adjusting for independent demographic predictors, and psychological factors were considered one at time, adjusting for independent demographic and social predictors. In the final model, variables that were no longer associated with the outcome at p < 0.10 were allowed to drop. We did not find colinearity to be a problem using this approach.

### Ethical considerations

The study obtained ethical approval from the Uganda Virus Research Institute’s Science and Ethics Committee, the Uganda National Council of Science and Technology and the London School of Hygiene Ethics Committee. Study participants were invited to consent after being provided with adequate information about the study. Respondents found to have significant psychiatric problems were referred to the psychiatric department at Entebbe district hospital for further assessment and management.

## Results

### Study population

From 6^th^ May 2010 to 10^th^ August 2010, 680 patients attending the HIV clinics at Entebbe hospital and Kigungu health centre were screened and given appointments to be interviewed for this study. Of these 618 (90.9 %) were eventually enrolled into the study while 62 (9.1 %) failed to turn up for their interview appointment despite telephone reminders. Of those who were enrolled into the study, Entebbe district hospital contributed 531 (85.9 %) while the smaller Kigungu Health Centre III contributed 87 (14.1 %). The two health facilities are located in the same district of Entebbe although the smaller health unit of Kigungu predominantly serves the fishing landing site of Kigungu while Entebbe hospital mainly serves the population in the urban centre. The study population of persons living with HIV/AIDS (PLWHA) was derived from the semi-urban and urban areas of Entebbe district who are better endowed socio-economically when compared with the general population of PLWHA in Uganda who are predominately from poor peasant agricultural backgrounds.

### Characteristics of suicidality

The prevalence of ‘moderate to high risk for suicidality’ (MHS) in this study was 7.8 % (95 % CI, 5.6 %- 9.9 %) and that of life-time attempted suicide was 3.9 % (95 % CI, 2.4 %- 5.4 %). The number of previous suicide attempts reported (n = 22) were: ‘one previous suicide attempt’ 17 (77.3 %), ‘two previous suicide attempts’ 3 (13.6 %), ‘three previous suicide attempts’ 1(4.5 %) and ‘five previous suicide attempts’ 1(4.5 %). On ‘methods of attempted suicide’ used during the most recent attempt (n = 21) these were: non-HIV medications 9 (42.9 %), HIV medications 2 (9.5 %), drowning 3 (14.3 %), organophosphate poisons 1 (4.8 %), hanging 1 (4.8 %), cutting 1 (4.8 %), jumping from a height 1 (4.8 %) and others not specified 3 (14.3 %).

### Socio-demographic correlates of MHS

Table 1 depicts the socio-demographic characteristics, with 449 (72.7 %) females, and 456 (73.8 %) patients in the 25–44 age band. Most (89.4 %) individuals had at least seven years of formal education but with only 53 (8.6 %) having gone on to attain a tertiary education. 275 (48.5 %) were married or currently living with someone, 75 (13.2 %) widowed and 132 (23.3 %) separated or divorced. Most (42.4 %) were employed as small scale tradespersons, artisans or in the transport business (taxi drivers, taxi conductors and boda boda riders-motorcycle taxis).

**Table 1:** Socio-demographic correlates of suicidality

	**Number in study (N,%)**	**Suicidality (n,%)**	**Unadjusted OR (95 % CI)**	**Adjusted OR (95 % CI)**^**1**^
**Sex**			**P = 0.003**	**P = 0.01**
Male	169 (27.3)	5 (3.0)	1	1
Female	449 (72.7)	43 (9.6)	3.47 (1.35-8.93)	3.06 (1.17-8.02)
**Age (years)**			**P = 0.17**	**P = 0.46**
19-24	59 (9.5)	5 (8.5)	1	1
25-34	238 (38.5)	25 (10.5)	1.27 (0.46-3.46)	1.30 (0.48-3.57)
35-44	218 (35.3)	11 (5.1)	0.57 (0.19-1.72)	0.72 (0.24-2.18)
45 +	103 (16.7)	7 (6.8)	0.79 (0.24-2.60)	1.00 (0.30-3.35)
**Marital status**			**P = 0.81**	**P = 0.64**
Currently married/				
cohabiting	275 (48.5)	24 (8.0)	1	1
Widowed	75 (13.2)	5 (6.0)	0.73 (0.27-1.98)	0.59 (0.20-1.71)
Separated/Divorced	132 (23.3)	13 (9.1)	1.14 (0.56-2.31)	0.96 (0.46-1.99)
Single	85 (15.0)	6 (6.4)	0.78 (0.31-1.99)	0.65 (0.25-1.67)
**Education level**			**P = 0.59**	**P = 0.52**
No education	65 (10.5)	7 (10.8)	1	1
Primary only	289 (46.8)	20 (6.9)	0.62 (0.25-1.52)	0.61 (0.24-1.53)
Secondary and				
above	264 (42.7)	21 (7.9)	0.72 (0.29-1.76)	0.80 (0.32-2.00)
**Religion**			**P = 0.28**	**P = 0.14**
Christian	535 (86.9)	44 (8.2)	1	1
Moslem	81 (13.1)	4 (4.9)	0.58 (0.20-1.66)	0.47 (0.16-1.37)
**Employment Status**			**P = 0.43**	**P = 0.80**
Farmer/Fisherman	97 (15.7)	6 (6.2)	1	1
Professional/Clerical	43 (7.0)	23(7.0)	1.14 (0.27-4.78)	1.06 (0.25-4.56)
Tradesperson/				
artisan/transport				
worker	262 (42.4)	20 (7.6)	1.25 (0.49-3.22)	0.96 (0.36-2.57)
Unemployed/house				
Wife	131 (21.2)	15 (11.4)	1.96 (0.73-5.25)	1.31 (0.46-3.75)
Others (including				
students)	84 (13.6)	4 (4.8)	0.76 (0.21-2.78)	0.66 (0.17-2.49)
**Clinic**			**P = 0.43**	**P = 0.39**
Entebbe distric				
hospital	531 (85.9 %)	43 (8.1 %)	1	1
Kigunga health centre	87 (14.1 %)	5 (5.7 %)	0.69 (0.27-1.80)	0.66 (0.25-1.73)

^1^ adjusted for age group and sex.

On socio-demographic factors (Table 1), it was only female gender adjusted Odds Ratio (aOR) 2.26, 95 % CI = 1.06-4.84, P = 0.01) that was significantly associated with MHS. Age, educational attainment, religion, marital status and occupation were not significantly associated with MHS. There were no differences in the prevalence of MHS by HIV clinic attended.

### Social factors associated with MHS

Table 2, majority 358 (57.9 %) lived more than 5 Km from the HIV clinic they were attending. Sixty nine (11.2 %) respondents reported food insecurity for themselves and their families. Majority 465 (75.6 %) had known their HIV status for 13 or more months, with most 399 (64.6 %) on ART. On negative life events experienced in the last 6 months, 350 (56.6 %) reported 1–5 events; 191 (43.7 %) reported 6–10 events; 77(12.5 %) reported 11+ events.

**Table 2:** Social correlates of suicidality

	**Number in study (N,%)**	**Suicidality (n,%)**	**Unadjusted OR (95 % CI)**	**Adjusted OR (95 % CI)**^**1**^
**Food Security**			**P = 0.04**	**P = 0.05**
Enough	549 (88.8)	38 (6.9)	1	1
Not enough	69 (11.2)	10 (14.5)	2.28 (1.08-4.81)	2.26 (1.06-4.84)
**Distance from HIV Clinic**			**P = 0.80**	**P = 0.76**
Less than 3 km	94 (15.2)	8 (8.5)	1	1
3 to 5 km	166 (26.9)	11 (6.6)	0.76 (0.30-1.99)	0.72 (0.28-1.89)
More than 5 km	358 (57.9)	29 (8.1)	0.95 (0.42-2.15)	0.91 (0.40-2.08)
**When knew HIV Status**			**P = 0.92**	**P = 0.88**
Up to 12 months ago	150 (24.4)	12 (8.0)	1	1
More than 12 months	465 (75.6)	36 (7.7)	1.03 (0.52-2.05)	0.95 (0.47-1.92)
**On ART**			**P = 0.75**	**P = 0.38**
Yes	399 (64.6)	32 (8.0)	1.10 (0.59-2.06)	1.34 (0.69-2.60)
No	219 (35.4)	16 (7.3)	1	1
**Social Support**			**P = 0.09**	**P = 0.19**
Low	188 (30.4)	20 (10.6)	1.71 (0.94-3.12)	1.52 (0.82-2.79)
High	430 (69.6)	28 (6.5)	1	1
**Negative life events**			**P < 0.001**	**P < 0.001**
1-5 events	350 (56.6)	11 (3.1)	1	1
6-10 events	191 (43.7)	16 (8.4)	2.82 (1.28-6.20)	2.63 (1.19-5.83)
11 +	77 (12.5)	21 (27.3)	11.6 (5.3-25.3)	10.6 (4.79-23.5)
**Stress Score index**			**P < 0.001**	**P < 0.001**
Low (score 0)	164 (26.5)	4 (2.4)	1	1
Medium (score 1–10)	323 (52.3)	16 (5.0)	2.08 (0.69-6.34)	1.95 (0.64-5.97)
High (score > 10)	131 (21.2)	28 (21.4)	10.9 (3.70-31.9)	9.69 (3.28-28.7)

^1^ adjusted for age group and sex.

After adjusting for age and sex, the social factors significantly associated with MHS were (Table 2): food insecurity (aOR 2.26, 95 % CI = 1.06-4.84); increasing number of negative life events experienced in the last 6 months, (aOR = 10.6, 95 % CI = 4.79-23.5, for those reporting 11+ events compared with those reporting 1–5 events) and increasing stress scores (aOR 9.69, 95 % CI = 3.28-28.7 for those with high scores compared with those with low stress scores). Factors not significantly associated with MHS were: ‘distance from HIV clinic’, ‘when got to know HIV status’, and ‘being on ART’ and social support.

### Psychological and clinical factors correlated with of MHS

Table 3 depicts the psychological and clinical factors, 16(2.6 %) had a past psychiatric history, while 120 (19.6 %) had a family history of psychiatric illness. 396 (64.1 %) had significant HIV associated neurocognitive impairment (a score of 10 or less on the International HIV Dementia Scale; Sacktor et al., 2005). The prevalence of psychiatric disorders/problems included major depressive disorder 50 (8.1 %), post traumatic stress disorder 10 (1.6 %), generalised anxiety disorder 5 (0.8 %) and alcohol dependency disorder 4 (0.7 %). On CD4 counts, 66 (12.5 %) had <100 cells/μL, 159 (30.2 %) had 100–249 cells/μL while 220 (35.6 %) had +350 cells/μL, the majority 387 (64.9 %) had a normal BMI.

**Table 3:** Psychological and Clinical correlates of suicidality

	**Number in study (N,%)**	**Suicidality (n,%)**	**Unadjusted OR (95 % CI)**	**Adjusted OR (95 % CI)**^**1**^
**Negative Coping Style index**			**P = 0.009**	**P = 0.02**
Low	101 (16.3)	5 (5.0)	1	1
Medium	322 (52.1)	18 (5.6)	1.14 (0.41-3.14)	1.08 (0.39-3.03)
High	195 (31.6)	25 (12.8)	2.82 (1.05-7.61)	2.54 (0.93-6.93)
**Past Psychiatric History**			**P = 0.03**	**P = 0.03**
Present	16 (2.6)	4 (25.0)	4.23 (1.31-13.7)	4.49 (1.33-15.1)
**Family history of psychiatric Illness**			**P = 0.08**	**P = 0.09**
Present	120 (19.6)	14 (11.7)	1.84 (0.95-3.56)	1.81 (0.92-3.54)
**Neurocognitive Impairment**			**P = 0.30**	**P = 0.29**
Present	396 (64.1)	34 (8.6)	1.39 (0.73-2.67)	1.41 (0.73-2.70)
**Psychosocial Impairment**			**P = 0.03**	**P = 0.03**
Present	318 (51.5)	32 (10.1)	1.99 (1.07-3.70)	2.01 (1.07-3.76)
**Most Recent CD4 count (cells/μL)**			**P = 0.37**	**P = 0.30**
< 100	66 (12.5)	4 (6.1)	1	1
100-249	159 (30.2)	15 (9.4)	1.61 (0.51-5.06)	1.52 (0.48-4.81)
250-349	81 (15.4)	9 (11.1)	1.94 (0.57-6.60)	1.69 (0.49-5.85)
350 +	220 (35.6)	13 (5.9)	0.97 (0.31-3.09)	0.81 (0.25-2.63)
**BMI Index**			**P = 0.60**	**P = 0.67**
Underweight	56 (9.2)	4 (7.1)	1	1
Normal	388 (64.9)	29 (7.5)	1.05 (0.35-3.11)	0.92 (0.30-2.78)
Overweight	117 (20.0)	13 (11.1)	1.62 (0.50-5.23)	1.30 (0.39-4.32)
Obese	35 (5.9)	2 (5.7)	0.79 (0.14-4.54)	0.57 (0.10-3.46)
**Diagnosis of post traumatic stress disorder**			**P < 0.001**	
Present	10 (1.6)	8 (80.0)	56.8 (11.7-276.4)	*
**Diagnosis of generalized anxiety disorder**			**P = 0.003**	
Present	5 (0.81)	3 (60.0)	18.9 (3.08-116.2)	*
**Alcohol dependency disorder**			**P = 0.29**	
Present	4 (0.65)	1 (25.0)	4.02 (0.41-39.4)	*
**Major depressive disorder**			**P < 0.001**	**P < 0.001**
Present	50 (8.1)	27 (54.0)	30.6 (15.1-62.0)	30.3 (14.4-63.8)

^1^ adjusted for age group and sex

* numbers too small to obtain reliable adjusted estimates.

**Table 4:** Final Multivariate Model of risk factors for suicidality in HIV/AIDS

	**Adjusted OR**
	**(95 % CI)**^**1**^
**Sex**	**P = 0.03**
Male	1
Female	2.86 (0.99-8.27)
**Negative life events**	**P = 0.009**
1-5 events	1
6-10 events	1.73 (0.72-4.16)
11 +	4.35 (1.72-11.0)
**Past Psychiatric Diagnosis**	**P = 0.04**
Present	5.34 (1.30-21.9)
**Major depressive disorder**	**P < 0.001**
Present	19.4 (9.00-41.7)

^1^ adjusted for all factors listed in the table.

Factors significantly associated with MHS were: increasing negative coping style index score (aOR 2.54, 95 % CI = 0.93–6.93, for those with a high score compared with low scores), a past history of psychiatric illness (aOR 4.49, 95 % CI = 1.33–15.1) psychosocial impairment (aOR 2.01, 95 % CI = 1.07–3.76), and a diagnosis of major depressive disorder, excluding suicidality items (aOR = 30.3, 95 % CI = 14.4–63.8). MHS was associated with post traumatic stress disorder and generalised anxiety disorder in the unadjusted analysis; however, the number with each diagnosis was too small (≤10) to obtain reliable estimates of the association adjusted for confounders.

Factors not significantly associated with MHS were a family history of psychiatric illness, HIV- associated neurocognitive impairment, CD4 count or BMI.

### Factors associated with MHS at multivariate analysis

In the final multivariable model, female sex, increasing number of negative life events, a past psychiatric history, and a diagnosis of major depressive disorder were independently associated with MHS. Adjusting for HIV clinic in the final model did not change any of the ORs appreciably, indicating that clinic was not an important confounder. Post traumatic stress disorder and generalised anxiety disorder were not considered for inclusion in the final model because the numbers were too small.

## Discussion

The paper sought to investigate the prevalence and the psychological, social and biological correlates of ‘moderate to high risk for suicidality’ (MHS) in HIV/AIDS in the African socio-cultural context. The principal finding of this study is that among ambulatory HIV/AIDS patients in the sub-Saharan African environment of Uganda, an increasing number of negative life events, past psychiatric history, and major depressive disorder were independent determinants of MHS. These results are in agreement with the stress-vulnerability model where social and psychological stressors acting on an underlying diathesis (including previous and current psychiatric morbidities) leads to suicidality [19].

The prevalence of a ‘moderate to high risk for suicidality’ (MHS) in this study was 7.8 %, a figure similar to that reported for suicidal ideation of 12.4 % by Kinyanda (1998) in urban Pre-ART Uganda, 13 % by Petruskin et al., (2005) in urban Post-ART Uganda and more recently of 8.8 % by Rukundo *(personal communication)* in semi-urban south-western Uganda. A life-time attempted suicide rate of 3.9 % reported in this study is similar to that of 3.1 % reported by Rukundo *(personal communication)* in semi-urban south-western Uganda but much lower than the rates reported in western studies [7-11]. Some of this difference with western studies can be attributed to differences in the risk for suicidality inherent to the sub-population being investigated [7,20]. The sub-populations at risk for HIV in the west (‘men who have sex with men’ and IV drug users) have an inherently increased risk for suicidality independent of there HIV serostatus, this contrasts with the lower risk for suicidality associated with the general population derived heterosexually married sub-population (in this study sample, 48.5 %) who now constitute the biggest risk category for new HIV infections in sub-Saharan Africa [20,21].

The majority of suicide attempters in this study (77.3 %) had done so once, similar results were reported by Kinyanda et al. (2005b) [22] among a general hospital sample of suicide attempters in urban Uganda where the rate for first time suicide attempters was 75 %. The main method of suicide attempt reported in this study was the use of medications (both HIV medications and others, 52.4 %), this contrasts with the findings of Kinyanda et al., (2004) [23] who among a general hospital sample reported that the main method of attempted suicide was by poisons (mainly organophosphates, 65 %). In this study female gender conferred a three fold increased risk for suicidality relative to the male gender, a similar female predominance has been reported in South Africa [24] and France [9] but has not been observed in other western countries [2,7].

Social factors associated with an increased risk for suicidality in this study included an increasing number of negative life events and the associated stress and food insecurity. Previous research has reported the following negative life events to be correlated with suicidality in HIV/AIDS: physical and sexual abuse, multiple HIV-related losses, loss of employment or insurance cover, financial difficulties, and partner relational problems [2,9,11,13]. Food insecurity has previously been correlated with major depressive disorder in HIV/AIDS [25].

The psychological factors of a past psychiatric history and a diagnosis of major depressive disorder were independent correlates of MHS in this study. Bellini and Bruschi (1996) [7] in a review of studies on HIV infection and suicidality pointed out that, ‘suicide attempts occur mainly in persons with a psychiatric history, previous attempted suicides or drug dependence’. More recent studies have also reported an association between suicidality in HIV/AIDS and psychological distress [2,11] and major depressive disorder [12,13,23].

Other psychological factors reported to be correlated with NHS in this study were a negative coping style and psychosocial impairment. Previous studies have reported that the positive coping style/adjustment of ‘spirituality’ and ‘a fighting spirit’ to be protective against suicidality [11,12,17]. Pugh and colleagues (1993) studying a case series of HIV positive suicide in London observed that worsening physical health was a risk factor for suicide in HIV/AIDS [26]. In this study there was no evidence for the role of the neurotoxic effect of the HIV virus and for HIV associated neurocognitive impairment as correlates of NHS.

Limitations of this study include firstly, that the cross sectional nature of this study made it difficult to determine the direction of causality of the investigated factors and MHS. Therefore, there is need for longitudinal studies to establish the exact causal direction between the investigated variables and MHS. Secondly, the number of individuals with some of the diagnosed psychiatric disorders was too small to enable us to satisfactorily explore independent associations with MHS. Thirdly, the threshold used as a cut off point for ‘moderate to high risk for suicidality’ (MHS) was derived from the authors of the M.I.N.I. neuropsychiatric assessment and has never been locally validated in the African socio-cultural environment. However the items used to assess for risk for suicidality i.e. previous suicide attempt, suicidal ideation, hopelessness and degree of planning have previously been shown to be associated with suicidality in the African environment by the first author [20].

Fourthly, the use of the surrogate measure “risk for suicidality” instead of “suicidality” as the dependent variable may have reduced the importance of the correlates identified in this study.

Lastly, a number of the tools used to assess various psychosocial constructs have not been locally validated. These tools were however locally adapted through a forward and backward translation process and to minimise bias, only those tools with a minimum α Cronbach of 0.50 were used in the analysis for this paper.

## Conclusions

These results are in agreement with the stress-vulnerability model where social and psychological stressors acting on an underlying diathesis (including previous and current psychiatric morbidities) leads to suicidality [19]. These results identify potential targets to mitigate risk through treatment of psychiatric disorders and promoting greater adaptation to living with HIV/AIDS [13]. Suicide prevention efforts in HIV/AIDS should therefore be directed at increased screening and treatment of psychiatric disorders in HIV/AIDS.

## Abbreviations

EK: Eugene Kinyanda; SH: Susan Hoskins; JN: Juliet Nakku; SN: Saira Nawaz; VP: Vikram Patel.

## Competing interests

The authors declare that they have no competing interests.

## Authors’ contributions

Concept: EK,SH, VP; Data collection: EK, SH, JN; Data analysis: EK, SN; First draft: EK, SH, JN, VP; Final revision: EK, SH, JN, VP, SN; All authors read and approved the final manuscript.

## Pre-publication history

The pre-publication history for this paper can be accessed here:

http://www.biomedcentral.com/1471-244X/12/63/prepub

## References

[B1] Lonnqvist J Wasserman D Physical illness and suicide Suicide-An unnecessary death 2001 Martin Dunitz, London, UK 93 98

[B2] Sherr L Lampe F Fisher M Arthur G Anderson J Zetler S Johnson M Edwards S Harding R Suicidal ideation in UK HIV clinic attenders AIDS 2008 22 1651 1658 10.1097/QAD.0b013e32830c4804 18670226

[B3] Kinyanda E Frequency with which psychiatric disorder is associated with a positive HIV-1 serostatus as seen in persons attending a TASO clinic in Mulago. Kampala, Uganda: Masters thesis 1998 Makerere University; . Department of Psychiatry,

[B4] Musisi S Kinyanda E Emotional and behavioural disorders in HIV seropositive adolescents in urban Uganda East Afr Med J 2009 86 1 16 24 19530544 10.4314/eamj.v86i1.46923

[B5] Petrushkin A Boardman J Ovuga E Psychiatric disorders in HIV- positive individuals in urban Uganda Psychiatr Bull 2005 29 55 458

[B6] Olley BO Zeier MD Seedat S Stein DJ Post-traumatic stress disorder among recently diagnosed patients with HIV/AIDS in South Africa AIDS Care 2005 17 5 550 557 10.1080/09540120412331319741 16036241

[B7] Bellini M Bruschi C HIV infection and suicidality J Affect Disord 1996 38 2–3 153 164 8791184 10.1016/0165-0327(96)00009-2

[B8] Kelly B Raphael B Judd F Kernutt G Burnett P Burrows G Suicidal ideation, suicide attempts, and HIV infection Psychosomatics 1998 39 405 415 10.1016/S0033-3182(98)71299-X 9775697

[B9] Preau M Bouhnik AD Peretti-Watel P Obadia Y Spire B on behalf of the ANRS-EN12-VESPA Group Suicide attempts among people living with HIV in France AIDS Care 2008 20 8 917 924 18777220 10.1080/09540120701777249

[B10] Simoni JM Nero DK Weinberg BA Suicide attempts among seropositive women in New York city Am J Psychiatry 1998 155 1631 1632 9812140

[B11] Cooperman NA Simoni JM Suicidal ideation and attempted suicide among women living with HIV/AIDS J Behav Med 2005 28 2 149 156 10.1007/s10865-005-3664-3 15957570

[B12] Lawrence ST Willig JH Crane HM Ye J Aban I Lober W Nevin CR Batey DS Mugavero MJ McCullumsmith C Wright C Kitahata M Raper JL Saag MS Schumacher JE Routine, self-administered, touch-screen, computer-based suicidal ideation assessment linked to automated response team notification in an HIV primary care setting Clin Infect Dis 2010 50 8 1165 1173 10.1086/651420 20210646 PMC2841210

[B13] McDaniel JS Brown L Goodkin K Cournos F Lyketsos C Working Group on HIV/AIDS Practice guideline for the treatment of patients with HIV/AIDS JAMA 2000 157 11 Suppl 1 62

[B14] Kerkhof AJFM Bernasco W Bille-Brahe U Platt S Schmidtke A Schiødt H, Aagaard B A WHO/EURO Multicentre study on parasuicide European Parasuicide study interview schedule. EPSIS I version 6.2 1989 Department of Clinical and Health Psychology, University of Leiden, The Netherlands

[B15] Kinyanda E Hjelmeland H Musisi S Life events in deliberate self-harm as seen in African population in Uganda Crisis 2005 26 1 4 11 15762078 10.1027/0227-5910.26.1.4

[B16] Sheehan D Lucrubier Y Sheehan KH Amorim P Janavs J Weiller E Herqueta T Baker R Dunbar GC The Mini-International Neuropsychiatric Interview (M.I.N.I.): the development and validation of a structured diagnostic psychiatric interview for DSM-IVand ICD-10 J Clin Psychiatry 1998 59 suppl 20 22 33 9881538

[B17] Watson M Greer S Young J Inayat Q Burgess C Robertson B The development of a questionnaire measure of adjustment to cancer: the MAC Psychol Med 1988 18 1 203 209 10.1017/S0033291700002026 3363039

[B18] Sacktor NC Wong M Nakasujja N Skolasky RL Selnes OA Musisi S Robertson K McArthur JC Ronald A Katibira E The International HIV Dementia Scale: A new rapid screening test for HIV dementia AIDS 2005 19 1367 1374 16103767

[B19] Mann JJ Waternaux C Haas G Malone K Towards a clinical model of suicidal behavior in psychiatric patients Am J Psychiatry 1999 156 181 189 9989552 10.1176/ajp.156.2.181

[B20] Marzuk PM Perry W Suicide and HIV: researchers and clinicians beware AIDS Care 1993 5 4 387 390 10.1080/09540129308258008 8110853

[B21] Shafer LA Nsubuga RN Seeley J Levin J Grosskurth H Examining the components of population-level sexual behaviour trends from 1993 to 2007 in an open Uganda cohort Sex Transm Dis 2011 38 12 697 704 21844720 10.1097/OLQ.0b013e318214e42e

[B22] Kinyanda E Hjelmeland H Musisi S Kigozi F Walugembe J Repetition of deliberate self-harm as seen in Uganda Arch Suicide Res 2005 9 333 344 10.1080/13811110500182208 16179329

[B23] Kinyanda E Hjelmeland H Musisi S Deliberate self-harm as seen in Kampala, Uganda Soc Psychiatry Psychiatr Epidemiol 2004 39 318 325 10.1007/s00127-004-0748-2 15085335

[B24] Schlebusch L Vawda N HIV-infection as a self-reported risk factor for attempted suicide in South Africa Afr J Psychiatry 2010 13 4 280 283 10.4314/ajpsy.v13i4.61877 20957327

[B25] Wu DY Munoz M Espiritu B Zeladita J Sanchez E Callacna M Rojas C Arevalo J Caldas A Shin S Burden of depression among impoverished HIV-positive women in Peru J Acquir Immune Defic Syndr 2008 48 4 500 504 10.1097/QAI.0b013e31817dc3e9 18614919

[B26] Pugh K O’Donnell I Catalan J Suicide and HIV disease AIDS Care 1993 5 4 391 400 10.1080/09540129308258009 8110854

